# Ego defense mechanisms in Pakistani medical students: a cross sectional analysis

**DOI:** 10.1186/1471-244X-10-12

**Published:** 2010-01-29

**Authors:** Maria A Parekh, Hina Majeed, Tuba R Khan, Anum B Khan, Salman Khalid, Nadia M Khwaja, Roha Khalid, Mohammad A Khan, Ibrahim M Rizqui, Imtiaz Jehan

**Affiliations:** 1 Department of Biological & Biomedical Sciences, Aga Khan University, Karachi, Pakistan; 2 Medical College, Aga Khan University, Karachi, Pakistan; 3 Department of Community Health Sciences, Aga Khan University, Karachi, Pakistan

## Abstract

**Background:**

Ego defense mechanisms (or factors), defined by Freud as unconscious resources used by the ego to reduce conflict between the id and superego, are a reflection of how an individual deals with conflict and stress. This study assesses the prevalence of various ego defense mechanisms employed by medical students of Karachi, which is a group with higher stress levels than the general population.

**Methods:**

A questionnaire based cross-sectional study was conducted on 682 students from five major medical colleges of Karachi over 4 weeks in November 2006. Ego defense mechanisms were assessed using the Defense Style Questionnaire (DSQ-40) individually and as grouped under Mature, Immature, and Neurotic factors.

**Results:**

Lower mean scores of Immature defense mechanisms (4.78) were identified than those for Neurotic (5.62) and Mature (5.60) mechanisms among medical students of Karachi. Immature mechanisms were more commonly employed by males whereas females employed more Neurotic mechanisms than males. Neurotic and Immature defenses were significantly more prevalent in first and second year students. Mature mechanisms were significantly higher in students enrolled in Government colleges than Private institutions (p < 0.05).

**Conclusions:**

Immature defense mechanisms were less commonly employed than Neurotic and Mature mechanisms among medical students of Karachi. The greater employment of Neurotic defenses may reflect greater stress levels than the general population. Employment of these mechanisms was associated with female gender, enrollment in a private medical college, and students enrolled in the first 2 years of medical school.

## Background

Ego defense mechanisms (or factors) were defined for the first time by Sigmund Freud as unconscious resources used by the *ego *to reduce the conflict between the *id *and the *superego *[1]. They provide a reflection of how an individual deals with conflict and stress, and thus, have been turned into the first psychoanalytical concept recognized by the DSM-IV as axes for future studies [2]. Ego defense mechanisms have been hypothesized to act as one set of mediators in the stress-illness relationship [3].

The 20 defense mechanisms identified by the Diagnostic and Statistical Manual (DSM) have been classified by Andrews [4] into: (a) four **mature**: sublimation, humor, anticipation and suppression; (b) four **neurotic**: undoing, pseudo-altruism, idealization, reaction formation; (c) twelve **immature**: projection, passive aggression, acting out, isolation, devaluation, autistic fantasy, denial, displacement, dissociation, splitting, rationalization and somatization. [see Additional file 1]

Vaillants' proposed Hierarchy of Defenses states that mature defense mechanisms are associated with better adaptive functioning and health, as opposed to immature defense which are correlated negatively with measures of adaptive adult functioning. Interestingly, neurotic defense mechanisms, despite being correlated with high levels of distress and impairment, have been seen to be protective in cognitive and affective awareness of conflicts, when compared to immature defenses [5,6].

Several studies have determined the association between individual defense mechanisms arising as a result of anxiety, and levels of adult functioning [7-12]. Their findings have been remarkably consistent in supporting the hierarchy of defenses, suggesting that instruments which could readily yield an accurate assessment of defensive functioning would prove clinically useful in identifying characteristic personality traits.

It is already widely accepted that medical students and physicians exhibit unique personality characteristics, which Shaw et al [13] suggest is due to the interaction of inherent personality patterns and specific environmental influences and stresses of medical school. Perceived medical school stress has been linked to clinically significant mental distress, which subsequently leads to mental health concerns such as anxiety and depression [14].

Several studies have been conducted in Karachi to assess the levels of stress and depression in medical students. Researchers investigating a similar population of medical students concluded that more than 90% of the students admit to being stressed at one time or another, with fourth and final year students showing a greater tendency than students of other years [15]. Results from a private medical university in Karachi have documented that 60% of its students suffer from anxiety and depression [16] while another study conducted at a government medical college reported a prevalence of 70% [17]. With a prevalence of 34% reported in the general population [18], these statistics clearly establish a greater stress level among Pakistani medical students. Numerous studies have reported the prevalence of ego defense mechanisms employed by medical students as a reaction to anxiety and depression. However, to the best of our knowledge, no such study has been conducted in Pakistan.

Our study aims to identify the prevalence of various ego defense mechanisms employed by medical students of Karachi. Although the current study does not directly assess the association between anxiety and ego defenses, it serves to highlight the various defense mechanisms commonly employed by a sub-population established to have greater stress levels, based on prior published literature, than the Pakistani general population. The data collected would aid in identifying the subset of medical students more likely to employ immature and neurotic defense mechanisms, which would then serve as a target for medical practitioners for teaching interventions, mental health-promoting strategies, and various awareness programs. The goal of these interventional programs would be to assist individuals in acquiring greater insight by bringing their unconscious behavior to consciousness and assisting them in understanding the cause of the behavior. This could eventually encourage adoption of mature defense mechanisms, and hence, a better quality of life, in coalition with social support systems, or psychotherapy.

## Methods

This descriptive study was a self-administered questionnaire-based cross-sectional analysis conducted over four weeks in November 2006.

### Subjects

A pilot study was initially conducted on 123 students of the Aga Khan University, which is the leading private medical college of Karachi, to assess if the DSQ-40 could be answered and interpreted by the students with ease. These results were not included in the final sample.

A simple random sample of 682 medical students was selected from 5 government and private institutes in Karachi, the country's largest and most populous city, to incorporate a large and representative sample. There are approximately equal numbers of government and private medical colleges and universities in Pakistan, all of which meet a strict criteria set by a central statutory and regulatory authority [19], ensuring a standard quality of education. However, private and government institutions differ in terms of subsidization of tuition fees and stronghold of student political unions, both greater in the latter.

There are 12 recognized medical colleges and universities in Karachi, with 90 to 200 students in every year [19]. Selection of the 5 medical colleges included in this study was based on simple random sampling. Students enrolled through Years 1 to 5 in the five major medical colleges of Karachi were included, irrespective of their age, sex, ethnicity, religion, or comorbids. Only those giving written consent were included in the study.

The exact number of medical students in Karachi or their gender distribution has not been reported. However, it is known that there are 12 recognized medical colleges and universities in Karachi, with 90 to 200 students in every year (Pakistan Medical & Dental Council). The estimated average number of students in each year is 160, hence making a total of 9600 medical students in the city of Karachi. This would mean that our study sample of 682 students represents approximately 7% of all medical students of Karachi, Pakistan.

### Methods of Measurement

Research on ego defense mechanisms has exponentially increased in recent years with the development of different tools for measuring defense mechanisms. The Defense Style Questionnaire (DSQ) is the most widely used self-report instrument for defense measurement with validated versions in numerous languages, including Chinese, Dutch, Arabic, Finnish, French, German, Italian, Japanese, and Norwegian [20]. This method has also been included in the American Psychiatric Association's Handbook of Psychiatric Measures (APA, 2000).

Bond et al initially developed the DSQ as an 88-item self-reported questionnaire to assess conscious derivatives of defense mechanisms [21], with the aim of identifying characteristic styles - conscious or unconscious - of how people deal with conflict, based on the idea that people can accurately comment on their behavior. In 1993, Andrews et al reorganized Vaillants' DSQ-67 into forty questions to make it short and manageable [4]. He classified the 20 defense mechanisms in accordance with the DSM-III-R into: (a) four **mature**: sublimation, humor, anticipation and suppression; (b) four **neurotic**: undoing, pseudo-altruism, idealization, reaction formation; (c) twelve **immature**: projection, passive aggression, acting out, isolation, devaluation, autistic fantasy, denial, displacement, dissociation, splitting, rationalization and somatisation.

The DSQ-40 is a reliable tool for assessing defense mechanisms [4,20,22,23]. The authors employed the 40 item DSQ-40 in English because it has enough questions to allow discrimination of defenses, is short enough to be used conveniently, and has results similar to the 67 item scale [4,20]. The DSQ-40 comprises of 40 items, used to derive scores on 20 defense mechanisms with two items for each defense mechanism, in a 9-point Likert format. The defense mechanisms are further grouped under three factors: a) Mature, b) Neurotic, and c) Immature style. [see Additional file 2]

### Data Analysis

The sample was grouped and compared based on gender, and year of medical education, with 1^st ^and 2^nd ^year students comprising the "preclinical group" while 3^rd^, 4^th ^and 5^th ^year students constituted the "clinical students group". Defense mechanisms employed by the students were also analyzed in relation to the 'type' of college they were enrolled in, i.e. private, or government medical colleges.

The DSQ-40 was analyzed in 2 ways as suggested by Andrews et al [4]: (a) by pairing 2 items together under the label of a defense mechanism; and (b) by grouping the defense mechanisms under Mature, Neurotic, and Immature.

Frequencies were calculated for categorical variables. The means (± standard deviation) for individual and grouped defenses were calculated. Respondents answered each of the 60 items on a 9 point Likert scale with anchors of one (not at all applicable to me) and nine (completely applicable to me). Scores for each defense were calculated by taking the mean of the two items representing the particular defense mechanism. Style scores were derived by taking the mean of the items belonging to each factor scale. Means were compared using the independent sample t-test. A p-value of less than 0.05 was considered to be statistically significant for all analyses. Only statistically significant results have been quoted in the current study.

### Ethical Approval, Informed Consent and Patient Confidentiality

This study complied with the Declaration of Helsinki. Patients who agreed to participate were explained the nature and the objectives of the study, and informed consent was formally obtained. The study was approved by the Ethical Review Committees of all participating medical institutes. No reference to the participant's identity was made at any stage during data analysis or in the paper.

## Results

The response rate (calculated from the number of missing questionnaires) was 96%.

Our study sample constituted 236 males (34.6%) and 446 females (65.4%). An adequate proportion was sampled from government (N = 418, 60.8%), and private (N = 269, 39.2%) medical colleges of Karachi, as was from preclinical (N = 366, 54.5%) and clinical (N = 306, 45.5%) groups of students. Mean age of the sample population was 20.18 years (16 to 26 years). Only 39 students (5.7%) of the total sample were aware of the concept of psychological defense mechanisms prior to this study.

Our study documents a lower mean score of immature ego defense mechanisms (4.78 ± 2.87) than that of mature (5.60 ± 2.56) and neurotic factors (5.62 ± 3.01). This was found to be statistically significant with a p value < 0.05 using independent sample t-test. The most common individual ego defense mechanisms employed by medical students were Rationalization (6.43 ± 2.34), Anticipation (6.34 ± 2.44), and Undoing (5.93 ± 2.67). The least commonly employed mechanisms were Devaluation (3.62 ± 2.52), Displacement (3.94 ± 2.87), and Denial (4.14 ± 2.79) (table 1).

**Table 1:** Prevalence of different Ego Defense Mechanisms, Full Sample

*Medical students (N = 682)*	
***Defense Mechanism***	***Mean (SD)***

***Mature***	**5.60 (2.56)**

Sublimation	5.08 (2.87)

Humor	5.74 (3.01)

Anticipation	6.34 (2.44)

Suppression	5.24 (3.02)

***Neurotic***	**5.62 (3.01)**

Undoing	5.93 (2.67)

Pseudoaltruism	5.82 (2.89)

Idealisation	5.45 (2.91)

Reaction Formation	5.11 (2.63)

***Immature***	**4.78 (2.87)**

Projection	4.68 (2.62)

Passive-aggression	4.40 (2.91)

Acting out	5.36 (2.77)

Isolation	5.12 (2.58)

Devaluation	3.62 (2.52)

Autistic fantasy	4.58 (2.71)

Denial	4.16 (2.79)

Displacement	3.94 (2.87)

Dissociation	4.71 (2.45)

Splitting	5.32 (2.56)

Rationalization	6.43 (2.34)

Somatization	4.95 (2.73)

### Gender Differences

**Figure 1:**
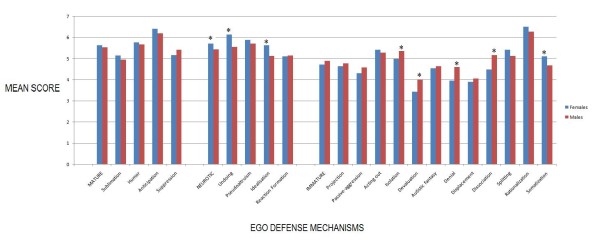
**Gender Differences in the Prevalence of EDM using Independent Sample T-test**.

There was no statistically significant difference in the prevalence of mature defense mechanisms amongst the two groups. However, the mean score of neurotic mechanisms was higher in females (5.72 vs 5.44; *p *< 0.05) than males. On comparison of individual defense mechanisms, the authors observed that Undoing, Idealization, and Somatization were significantly more prevalent in female medical students, whereas Isolation, Devaluation, Denial, and Dissociation were commonly employed by the male population (Figure 1)

* *Statistically Significant*

### Year of Medical Education

As mentioned above students were classified into 2 groups based on the year of their medical education: (a) Preclinical - Years 1 and 2; and (b) Clinical - Years 3, 4 and 5.

On comparison of the two groups, difference in mature factor failed to reach statistical significance. However, as hypothesized by the authors, neurotic (5.85 *vs *5.37; *p *< 0.05) and immature (4.88 *vs *4.67; *p *< 0.05) defense mechanisms were more commonly employed by students in the Preclinical than in the Clinical group.

Individual analysis of the ego defense mechanisms revealed that Idealization (5.69 *vs *5.20; *p *< 0.05), Reaction formation (5.35 *vs *4.87; *p *< 0.05), Splitting (5.67 *vs *4.93; *p *< 0.05), and Rationalization (6.59 *vs *6.26; *p *< 0.05) were more prevalent in preclinical students, while Undoing (5.68 *vs *5.16; *p *< 0.05) was the only defense mechanism significantly more prevalent amongst the clinical group.

The authors observed rationalization, which is an immature factor, to be more common among first and second year students than those in their clinical years (6.59 *vs *6.26, *p *< 0.05).

### Medical Institutions

Mean score of mature defense mechanisms was significantly higher in government medical colleges (5.68 vs 5.48; *p *< 0.05) as compared to students enrolled in private medical colleges. No difference was found in the means of Neurotic and Immature defense mechanisms between the two groups.

Anticipation (6.56 vs 6.01; *p *< 0.05) and Rationalization (6.54 vs 6.25; *p *< 0.05) were more common amongst students of government colleges while a larger proportion of students enrolled in private institutions employed Devaluation (3.87 vs 3.47; *p *< 0.05) and Displacement (4.15 vs 3.81; *p *< 0.05) defense mechanisms.

## Discussion

To our knowledge, this is the first study of its kind from Pakistan investigating the prevalence of ego defense mechanisms employed by medical students. Two major studies with similar objectives employing the DSQ-40 have been previously published [24,25].

The three main themes, reflecting the most prevalent mechanisms, representative of the characteristics of the Pakistani medical student population were:

### 1. Rationalization

Rationalization, defined by the DSM as, 'offering a socially acceptable and apparently logical explanation for an act or decision actually produced by unconscious impulses', was the most commonly employed ego defense mechanism by the participating medical students. The authors observed rationalization, which is an immature factor, to be more common among first and second year students than those in their clinical years. This finding may be explained by a study assessing the developmental aspects of psychological defenses which indicates that defense mechanisms tend to mature with advancement of age in adolescents to young adults [26].

### 2. Anticipation

Defined as 'anticipating consequences of possible future events and considering realistic, alternative responses or solutions', this was found to be the second most common ego defense mechanism with a mean score of 6.34 ± 2.44. A similar observation has been reported by La Cour [24] in Danish medical students. It was found to be so independent of gender and year of education. However, there was significant difference between private and government institutions, with Anticipation more commonly employed by government college students. As mentioned above, government institutions offer greater subsidization of tuition fees, catering more widely to students with a lower socioeconomic status. We may suggest that these students may be forced to face the harsh realities of life sooner than their rich counterparts.

### 3. Undoing

Before undertaking the current study, the authors hypothesized that Undoing was a rarely employed neurotic defense. However, it was observed to be the third most prevalent mechanism with a mean score of 5.93 ± 2.67, and was employed more commonly by females and students of the clinical group. In the questionnaire, it deals with undoing of aggressive behavior (e.g., Item 32: After I fight for my rights, I tend to apologize for my assertiveness). This finding may be explained by the 'hierarchical' nature of the extremely 'competitive' medical profession where "Undoing could be seen as a submissive, but adaptive, strategy in the field of competition. The 'fight for rights' is not a suitable attitude in an authoritarian environment", La Cour [24] aptly summarizes.

### Clinical vs. Preclinical

In this study, the authors observed a greater prevalence of immature and neurotic factors amongst students in their preclinical years. The general maturity of the psychological defense mechanisms is found to change over a lifespan [5]. In this study, however, there is only a minor age difference between the two groups. Hence, the inference that defense mechanisms mature with age and added responsibility in clinical years may be overzealous.

The differences in the prevalence of these defenses may then be explained by the increased levels of stress and the overall psychological maturity in senior medical students. Chronically persistent high levels of stress coupled with greater exposure and general maturation of the psyche may reflect the higher utilization of mature defense styles in this group of students.

A study conducted in Karachi investigating a similar population of medical students concluded that fourth and final year students showed a greater tendency to feel stressed (95% and 98% respectively) than students of other years [15]. Preliminary data of one particular study [27] assessing the level of distress in medical students in a pre-exam and normal school setting support the view that, upon entering medical school, students' emotional status resembles that of the general population. However, the rise in depression scores and their persistence over time suggest that emotional distress during medical school is chronic and persistent rather than episodic.

Among individual neurotic ego defense mechanisms, the mean scores of Undoing, Reaction formation and Idealization were significantly higher in students enrolled in their preclinical years. Splitting and Rationalization were the most prevalent immature defenses in this group. These findings may be explained, albeit cautiously, by the fact that Pakistani first and second year medical students are teenagers aged 17 to 19, at a point where their personalities are being molded and various life events tend to leave very strong impressions. These impressions are either completely positive or negative, and it is only with time and maturity that they learn to see the grayer shades of life.

### Gender Differences

Neurotic mechanisms were found to be more commonly employed by females, whereas the use of immature defenses was prevalent among male medical students.

The positing of gender differences in defenses on the basis of classical psychoanalytic theory [1] has generally been supported in prior investigations, which state that women tend to use internalizing defenses such as Introversion, and men are more likely to employ externalizing ones [28-30] such as Projection and Aggression [30-32].

In our study, the mean score of Isolation was found to be significantly greater in males than females. This is consistent with findings made by Watson and Sinha [25] and La Cour [24]. Females are generally more emotionally labile as compared to their male counterparts who are better at splitting emotional components from their thoughts [28], as shown by higher means for Isolation in men in our study.

The authors observed that female students employed Somatization more commonly than male students; also reported by La Cour [24]. These higher prevalence rates may be explained by women's greater psychological awareness of their bodily functions and reactions. Gender variations were also found in Undoing, Devaluation and Idealization defense mechanisms; however, these variations were inconsistent with those reported in prior studies.

Andrews et al [33] did not find any differences in the ego defense mechanism employed by the two genders. Studies by Watson, Sinha and La Cour, along with our own results do not support his findings. The authors support the suggestion made by Watson and Sinha that specific norms of the DSQ-40 need to be reconstructed with regards to gender.

### Limitations

Ego defense mechanisms are unconscious processes [1,6], and thus are not obviously amenable to measurement using self-report questionnaires. Nonetheless, they manifest as 'typical behaviors' in response to stress, which an individual is capable of reporting, [21] even if they lack insight into the defensive function of that behavior [34]

Even though the DSQ is a self-reporting tool offering portability, affordability, and quantification, it faces significant challenges in the domains of reliability and validity by virtue of the intrinsic complexity of defense mechanisms [35]. The DSQ-40, which has been used in a number of studies along similar themes, still remains under investigation. Trijsburg and colleagues [23] have emphasized that the validity of specific defenses, as demonstrated in this present study, is weak and that evidence for classifying defenses using this tool into immature, neurotic, and mature types is lacking. However, the DSQ, they conclude, remains a useful instrument for determining overall defensive functioning.

Subsequent to prior numerous revisions [4,23,31,33], recent efforts to improve the reliability, validity, and congruency of the tool with the DSM-IV resulted in the development of the DSQ-60 [36]. However, preliminary results show that the psychometric properties of the scale remain inadequate for broad use recommendation.

There is a lack of normative data on ego defense mechanisms employed by the general population of Karachi. As our data was collected from five different medical colleges of Karachi, it would be safe to say that our results can be generalized to represent the entire medical student population of Karachi, if not Pakistan. The overrepresentation of women in the sample is a simple reflection of the fact that an increasing percentage of medical students worldwide are female [37-39], and that a large proportion of the future physicians of Pakistan will be females. This highlights the importance of identifying the differences in psychological defense mechanisms employed by the two gender groups.

Our cross-sectional study design was limited in several aspects. Although this method is simple, convenient, and economically feasible, a temporal or causal relationship between stress levels and ego defense mechanisms can not be established based on these results. Furthermore, it is imperative to remember that the current study focused on medical students only, and hence, the results may not be applicable to the general population.

## Conclusions

The first psychoanalytical concept of the DSM based on Ego Defense Mechanisms, described by Sigmund Freud, provide a reflection of how an individual deals with conflict and stress. In this article, the authors identify and highlight the lower mean scores of immature defense mechanisms than those for neurotic and mature mechanisms among medical students of Karachi. The greater employment of these neurotic defenses may reflect greater stress levels than the general population. However, this must be interpreted with caution as there is no normative data on the prevalence of defense mechanisms in the general population of Karachi. In conclusion, we found female gender, enrollment in a private medical college, and preclinical years to be the strongest factors associated with the employment of neurotic mechanisms amongst Pakistani medical students. Future studies are recommended to confirm our findings, as the primary utilization of neurotic mechanisms as the basic style of coping predicts significant long-term maladaptive functioning, dissatisfaction, and poor quality of life. These factors are a useful resource for medical practitioners and student counselors to help identify students who are most in need of interventions to encourage adoption of mature defense mechanisms, and hence, attain a better quality of life. The findings of this study may suggest a need for further research to assess the effect of specific ego defense mechanisms employed by individuals on their quality of life.

## Competing interests

The authors declare that they have no competing interests.

## Authors' contributions

MAP and HM participated in the design of the study, performed the statistical analysis, and wrote the final manuscript. TRK, ABK, NMK and RK conceived the study, and participated in its design and coordination and helped to draft the manuscript. SK, MAK, IJ and IMR revised the manuscript for important intellectual content. All authors participated in data collection, read and approved the final manuscript.

## Pre-publication history

The pre-publication history for this paper can be accessed here:

http://www.biomedcentral.com/1471-244X/10/12/prepub

## Supplementary Material

Additional file 1 **Appendix 1**. DSM classification of ego defense mechanisms Click here for file

Additional file 2 **Appendix 2**. DSQ-40 Questionnaire Click here for file

## References

[B1] Freud A The Ego and the Mechanisms of Defence 1937 London: Hogarth Press and Institute of Psycho-Analysis

[B2] Perry JC Hoglend P Shear K Vaillant GE Horowitz M Kardos ME Bille H Kagan D Field trial of a diagnostic axis for defense mechanisms for DSM-IV J Personality Disorders 1998 12 56 68 10.1521/pedi.1998.12.1.56 9573520

[B3] Flannery RB Jr Towards stress resistant persons: a stress management approach to the treatment of anxiety American Journal of Preventive Medicine 1987 3 25 30 3330657

[B4] Andrews G Defense style questionnaire J Nerv Ment Dis 1993 181 4 246 56 10.1097/00005053-199304000-00006 8473876

[B5] Vaillant GE Adaptation to Life 1977 Boston, Little, Brown

[B6] Vaillant GE Battista JR Empirical test of Vaillant's hierarchy of ego functions Am J Psychiatry 1982 139 356 357 7058952 10.1176/ajp.139.3.356

[B7] Jacobsen AM Beardslee W Hauser ST Noam GG Powers SI Houlihan J Rider E Evaluating ego defense mechanisms using clinical interviews: an empirical study of adolescent diabetic and psychiatric patients J Adolesc 1986 9 303 319 10.1016/S0140-1971(86)80038-0 3805435

[B8] Perry JC Cooper SH Vaillant GE What do cross-sectional measures of defenses predict? Empirical Studies of Ego Mechanisms of Defense 1986 Washington, DC, American Psychiatric Press 32 40

[B9] Perry JC Cooper SH A preliminary report on defenses and conflicts associated with borderline personality disorder J Am Psychoanal Assoc 1986 34 863 893 10.1177/000306518603400405 3819306

[B10] Vaillant GE Natural history of male psychological health: the relation of choice of ego mechanisms of defense to adult adjustment Arch Gen Psychiatry 1976 33 535 545 1267569 10.1001/archpsyc.1976.01770050003001

[B11] Vaillant GE Bond M Vaillant CO An empirically validated hierarchy of defense mechanisms Arch Gen Psychiatry 1986 43 786 794 3729674 10.1001/archpsyc.1986.01800080072010

[B12] Vaillant GE Drake RE Maturity of ego defense in relation to DSM-III Axis II personality disorders Arch Gen Psychiatry 1985 42 597 601 4004502 10.1001/archpsyc.1985.01790290079009

[B13] Shaw DL Wedding D Zeldow P Diehl N Wedding D Special problems of medical students Behavior and medicine 2001 Seattle: Hogrefe & Huber 66 84

[B14] Dyrbye LN Thomas MR Shanafelt TD Systematic review of depression, anxiety, and other indicators of psychological distress among U.S. and Canadian medical students Acad Med 2006 81 4 354 73 10.1097/00001888-200604000-00009 16565188

[B15] Shaikh B Kahloon A Kazmi M Khalid H Nawaz K Khan N Khan S Students, Stress and Coping Strategies: A Case of Pakistani Medical School Educ Health (Abingdon) 2004 17 3 346 53 10.1080/13576280400002585 15848822

[B16] Inam SNB Saqib A Alam E Prevalence of Anxiety and Depression among Medical Students of Private University J Pak Med Assoc 2003 53 2 12705482

[B17] Khan MS Mahmood S Badshah A Ali SU Jamal Y Prevalence of depression, anxiety and their associated factors among medical students in Karachi, Pakistan J Pak Med Assoc 2006 56 12 583 6 17312648

[B18] Mirza I Jenkins R Risk factors, prevalence, and treatment of anxiety and depressive disorders in Pakistan: systematic review BMJ 2004 328 7443 794 10.1136/bmj.328.7443.794 15070634 PMC383372

[B19] Pakistan Medical & Dental Council, Regulations for the Degree of Bachelor of Medicine and Bachelor of Surgery http://dev.plexushosting.com/PMDC/Regulation/RegulationsForMBBS/tabid/114/Default.aspx

[B20] Bond M Perry JC Long-term changes in defense style with psychodynamic psychotherapy for depressive, anxiety and personality disorders American Journal of Psychiatry 2004 161 1665 1671 10.1176/appi.ajp.161.9.1665 15337658

[B21] Bond M Gardner ST Christian J Sigal JJ Empirical study of self-rated defense styles Arch Gen Psychiatry 1983 40 3 333 8 6830412 10.1001/archpsyc.1983.01790030103013

[B22] Yilmaz N Gençöz T Ak M Psychometric properties of the defense style questionnaire: a reliability and validity study Turk Psikiyatri Derg 2007 Fall, 18 3 244 53 17853979

[B23] Trijsburg RW Van t Spijker A Van HL Hesselink AJ Duivenvoorden HJ Measuring overall defensive functioning with the defense style questionnaire: a comparison of different scoring methods Journal of Nervous and Mental Disorders 2000 188 432 439 10.1097/00005053-200007000-00007 10919702

[B24] La Cour P Psychological Defenses of Danish Medical Students J Nerv Ment Dis 2002 190 22 26 10.1097/00005053-200201000-00006 11838026

[B25] Watson DC Sinha K Gender, Age, and Cultural Differences in the Defense Style Questionnaire-40 J Clin Psychol 1998 54 67 75 10.1002/(SICI)1097-4679(199801)54:1<67::AID-JCLP8>3.0.CO;2-R 9476710

[B26] Evans DW Seaman JL Developmental aspects of psychological defenses: their relation to self-complexity, self-perception, and symptomatology in adolescents Child Psychiatry Hum Dev 2000 Summer, 30 4 237 54 10.1023/B:CHUD.0000037152.88369.3a 10921207

[B27] Rosal MC Ockene IS Ockene JK Barrett SV Ma Y Hebert JR A longitudinal study of student's depression at one medical school Acad Med 1997 72 6 542 6 10.1097/00001888-199706000-00022 9200590

[B28] Levitt DB Gender differences in ego defenses in adolescence: sex roles as one way to understand the differences J Pers Soc Psychol 1991 61 6 992 9 10.1037/0022-3514.61.6.992 1774636

[B29] Brody L Rozek MK Muten E Age, sex and individual differences in children's defensive styles Journal of Clinical Child Psychology 1985 14 132 138 10.1207/s15374424jccp1402_6

[B30] Cramer P Defense mechanisms in adolescence Developmental Psychology 1979 15 476 477 10.1037/h0078084

[B31] Bond M Perry JC Gautier M Goldenberg M Oppenheimer U Simand J Validating the self-report of defense styles Journal of Personality Disorders 1989 3 101 112

[B32] Borrelli Scott E The differential use of ego defense mechanisms in early and late adolescence Dissertation Abstracts International 1979 40 5-B 2393

[B33] Andrews G Pollock C Stewart G The determination of defense style by questionnaire Archives of General Psychiatry 1989 46 455 460 2785372 10.1001/archpsyc.1989.01810050069011

[B34] Plutchik R Kellerman H Conte HR Izard CE A structural theory of ego defense and emotions Emotions in personality and psychopathology 1979 New York: Plenum Press 229 257

[B35] Endler NS Parker JDA Butcher JN Assessing a patient's ability to cope Practical considerations in clinical personality assessment 1996 New York: Oxford University Press 329 352

[B36] Thygesen K Drapeau M Trijsburg RW Lecours S de Roten Y Assessing defense styles: Factor structure and psychometric properties of the new Defense Style Questionnaire 60 (DSQ-60) International Journal of Psychology and Psychology Therapy 2008 8 2 171 181

[B37] Burton KR Wong IK A force to contend with: the gender gap closes in Canadian medical schools CMAJ 2004 170 1385 6 15111465 10.1503/cmaj.1040354 PMC395806

[B38] Barzansky B Etzel SI Medical schools in the United States, 2006-2007 JAMA 2007 298 1071 7 10.1001/jama.298.9.1071 17785656

[B39] BMA Board of Medical Education The demography of medical schools: a discussion document. London 2004

