# Supplementary material for: Ego defense mechanisms in Pakistani medical students: a cross sectional analysis
Source: BMC Psychiatry. 2010 Jan 29;10:12. doi: 10.1186/1471-244X-10-12 (PMC2836996; doi:10.1186/1471-244X-10-12)
Supplement: Additional file 1 — Appendix 1. DSM classification of ego defense mechanisms [file 1471-244X-10-12-S1.DOC]

**Appendix I: DSM Classification of Ego Defense Mechanisms:**

- **Sublimation:** redirecting 'wrong' urges into socially acceptable actions
- **Humor:** dealing with emotional conflict or external stressors by emphasizing the amusing or ironic aspects of the conflict or stressors
- **Anticipation:** anticipating consequences of possible future events and considering realistic, alternative responses or solutions
- **Suppression:** intentional exclusion of material from consciousness
- **Undoing:** an act or communication which partially negates a previous one
- **Pseudo-altruism:** deals with emotional conflict or internal or external stressors by dedication to meeting the needs of others
- **Idealization:** Overestimation of desirability & underestimation of limitations of desired object
- **Reaction Formation:** overacting in the opposite way to the fear
- **Projection:** attributing uncomfortable feelings to others
- **Passive Aggression:** deals with emotional conflict or internal or external stressors by indirectly and unassertively expressing aggression toward others
- **Acting Out:** dealing with emotional conflict or internal or external stressors by actions rather than reflections or feelings
- **Isolation:** splitting-off of the emotional components from a thought
- **Devaluation:** dealing with emotional conflict or internal or external stressors by attributing exaggerated negative qualities to self or others
- **Autistic fantasy:** deals with emotional conflict or internal or external stressors by excessive daydreaming as a substitute for human relationships, more effective action, or problem solving
- **Denial:** claiming/believing that what is true to be actually false.
- **Displacement:** redirecting emotions to a substitute target.
- **Dissociation:** Splitting-off a group of thoughts or activities from the main portion of consciousness; compartmentalization
- **Splitting:** other individuals or the self is perceived as "All good or all bad.
- **Rationalization:** Offering a socially acceptable and apparently more or less logical explanation for an act or decision actually produced by unconscious impulses
- **Somatisation:** Conflicts are represented by physical symptoms involving parts of the body innervated by the sympathetic and parasympathetic system
