# Supplementary material for: Ego defense mechanisms in Pakistani medical students: a cross sectional analysis
Source: BMC Psychiatry. 2010 Jan 29;10:12. doi: 10.1186/1471-244X-10-12 (PMC2836996; doi:10.1186/1471-244X-10-12)
Supplement: Additional file 2 — Appendix 2. DSQ-40 Questionnaire [file 1471-244X-10-12-S2.DOC]

**Appendix III: DSQ-40:**

**Rate each question on a scale of 1 (completely disagree) – 9 (fully agree):**

1. I get satisfaction from helping others and if this were taken away from me I would get depressed.

2. I am able to keep a problem out of my mind until I have time to deal with it.

3. I work out my anxiety through doing something constructive and creative like painting and woodwork.

4. I am able to find good reasons for everything I do.

5. I am able to laugh at myself pretty easily.

6. People tend to mistreat me.

7. If someone mugged me and stole my money, I’d rather he be helped than punished.

8. People say I tend to ignore the unpleasant facts as if they didn’t exist.

9. I ignore danger as if I were Superman.

10. I pride myself on my ability to cut people down to size.

11. I often act impulsively when something is bothering me.

12. I get physically ill when things aren’t going well for me.

13. I am a very inhibited person.

14. I get more satisfaction from my fantasies than from my real life.

15. I have special talents that allow me to go thought life without problems.

16. There are always good reasons when things don’t work out for me.

17. I work more things out in my daydreams than in my real life.

18. I fear nothing.

19. Sometimes I think I am an Angel and other times I think I am a devil.

20. I get openly aggressive when I feel hurt.

21. I always feel that someone I know is like a guardian Angel.

22. As far as I am concerned, people are either good or bad.

23. If my boss bugged me, I might make a mistake in my work or work more slowly so as to get back at him.

24. There is someone I know who can do anything and who is absolutely fair and just.

25. I can keep the lid on my feelings if they letting them out would interfere with what I am doing.

26. I am usually able to see the funny side of an otherwise painful predicament.

27. I get a headache when I have to do something I don’t like.

28. I often find myself being very nice to people who by all rights I should be angry at.

29. I am sure I get a raw deal from life.

30. When I have to face a difficult situation I try to imagine what it will be like and plan ways to cope with it.

31. Doctors never really understand what’s wrong with me.

32. After I fight for my rights, I tend to apologize for my assertiveness.

33. When I am depressed or anxious, eating makes me feel better.

34. I am often told that I don’t show my feelings.

35. If I can predict that I am going to be sad ahead of time, I can cope better.

36. No matter how much I complain, I never get a satisfactory response.

37. Often I find that I don’t feel anything when the situation would seem to warrant strong emotions.

38. Sticking to the task at hand keeps me from feeling depressed or anxious.

39. If I were in a crisis, I would seek out another person who had the same problem.

40. If I have an aggressive thought, I feel the need to do something to compensate for it.
